# Carrimycin suppresses the TCF3-BCR signaling axis and induces oxidative DNA damage in Burkitt lymphoma

**DOI:** 10.1007/s10495-026-02477-x

**Published:** 2026-11-11

**Authors:** Qingfeng Xue, Juan Ye, Herui Wang, Han Ye, Yaping Zhang, Shuran Chen, Zhengping Zhuang

**Affiliations:** Neuro-Oncology Branch, Center for Cancer Research, National Cancer Institute, National Institutes of Health, Bethesda, MD USA

**Keywords:** Carrimycin, Burkitt lymphoma, Reactive oxygen species, B-cell receptor signaling, Transcription factor 3, Central nervous system

## Abstract

Burkitt lymphoma (BL) is a highly aggressive B-cell malignancy with poor outcomes in relapsed or central nervous system (CNS)-involved cases. This study investigates the antitumor mechanism of Carrimycin, a genetically engineered macrolide antibiotic, in BL. In vitro assays demonstrated that Carrimycin exerts potent, dose-dependent cytotoxicity, inducing G0/G1 cell cycle arrest and caspase-dependent apoptosis. Mechanistically, it triggered the generation of reactive oxygen species (ROS), leading to DNA damage. Transcriptomic profiling and molecular assays revealed that Carrimycin binds to the transcription factor 3 (TCF3), causing a collapse of the tonic B-cell receptor (BCR) signaling pathway. Genetic knockdown of TCF3 partially rescued BL cells from Carrimycin-induced cytotoxicity, confirming this molecular dependency. In vivo, Carrimycin suppressed tumor burden and prolonged survival in both subcutaneous and intracranial models with a favorable safety profile. In conclusion, Carrimycin is a therapeutic agent that targets TCF3 to inhibit the tonic BCR signaling axis while simultaneously driving lethal oxidative stress. Its systemic and intracranial efficacy positions Carrimycin as a highly promising candidate for clinical translation in high-risk and CNS-involved BL.

**Supplementary Information:**

The online version contains supplementary material available at https://doi.org/10.1007/s10495-026-02477-x.

## Background

Burkitt lymphoma (BL) is a highly aggressive, MYC-driven mature B-cell malignancy characterized by one of the fastest proliferation rates among human cancers [1]. It is defined by the recurrent translocation t(8;14)(q24;q32), which places MYC under immunoglobulin enhancer control, driving constitutive overexpression and profound metabolic reprogramming [2, 3]. Although intensive chemotherapy is often curative, therapeutic failure occurs in a subset of high-risk patients [4–6]. Patients with relapsed or refractory (R/R) BL have a median overall survival of approximately 3 months, and those with central nervous system (CNS) involvement face a median survival of only 2–8 months [7, 8]. Therefore, it is critical to explore new therapeutic approaches to improve BL patient outcomes, especially for patients with R/R disease or CNS involvement.

B-cell receptor (BCR) signaling is essential for B-cell development and plays a central role in the pathogenesis of B-cell lymphomas [9]. The BCR apparatus transmits signals through two distinct modes: antigen-dependent ‘active’ signaling, engaging downstream effectors such as Bruton’s tyrosine kinase (BTK), and antigen-independent 'tonic' signaling, predominantly mediated via the PI3K-AKT pathway [10]. In BL, tonic signaling predominates and is essential for maintaining tumor cell viability. Consistently, preclinical studies have shown that phosphatidylinositol 3-kinase (PI3K) inhibition suppresses BL growth [11]. Despite the predominance of PI3K signaling, studies show that the BTK inhibitor ibrutinib also exerts significant anti-tumor activity in BL mouse models, suggesting a broader dependence on the BCR signalosome [12].

The E-protein family of basic helix-loop-helix (bHLH) transcription factors regulates stages of hematopoietic lineage specification [13]. Within this family, TCF3 (yielding the E12 and E47 splice variants) is strictly indispensable for early B-cell commitment, operating synergistically with early B-cell factor 1 (EBF1) to initiate B-lineage transcriptional programs [14]. The absolute necessity of this regulatory network is highlighted by murine knockout models; genetic ablation of either the entire *Tcf3* locus or specifically the E47 isoform induces a developmental arrest at the early pre-pro-B cell stage, underscoring its obligate role in normal lymphopoiesis [15]. Beyond its physiological role in early B cell development, dysregulated TCF3 activity is a hallmark oncogenic driver in BL. TCF3 dependency is firmly established in the pathogenesis of BL, where it is essential for tumor cell survival and the amplification of BCR signaling. Genomic profiling reveals that approximately 70% of sporadic BL cases harbor mutually exclusive mutations directly affecting *TCF3* or its endogenous negative regulator, *ID3* [16]. These mutually exclusive alterations culminate in hyperactive TCF3 signaling, which enforces a critical dependency loop in these tumors. Mechanistically, sustained TCF3 activity augments tonic BCR signaling and subsequently hyperactivates the pro-survival PI3K cascade, establishing a vital and targetable vulnerability in BL [16].

To address the therapeutic challenges associated with BL, novel targeted agents are actively being explored. Carrimycin, a macrolide antibiotic composed of Isovalerylspiramycin (ISP) I, II, and III, was previously mainly used for anti-infection purposes [17]. However, emerging evidence has repositioned it as a promising anti-tumor candidate, with demonstrated efficacy against oral squamous cell, lung, and hepatocellular carcinomas [18–20]. Notably, our previous work revealed a distinct mechanism of action: ISP I targets nuclear selenoprotein H to induce accumulation of reactive oxygen species (ROS), thereby suppressing tumor growth and metastasis [21]. Despite these encouraging findings in solid tumors, the therapeutic potential of Carrimycin in BL remains unexplored.

In this study, we investigated the therapeutic efficacy and underlying molecular mechanisms of Carrimycin in BL. We showed that Carrimycin exerts potent anti-tumor activity both in vitro and in vivo by blocking the BCR signaling network through the binding and degradation of TCF3. This targeted disruption is coupled with ROS-induced DNA damage, culminating in tumor cell apoptosis and cell cycle arrest. Crucially, Carrimycin demonstrates efficacy in an intracranial xenograft model, suggesting its capacity to treat CNS-involved disease. Ultimately, these data establish Carrimycin as a multi-mechanistic therapeutic agent, providing a preclinical foundation for its advancement into clinical trials for refractory or CNS-involved BL.

## Methods

### Cell lines and cell viability assay

Burkitt lymphoma (Raji, NAMALWA, CA46, Ramos), anaplastic large-cell lymphoma (ALCL; KARPAS 299, SR-786), and diffuse large B-cell lymphoma (DLBCL; OCI-LY10, U-2932, TMD8) cell lines were purchased from the ATCC database and were cultured in RPMI-1640 medium supplemented with 10% fetal bovine serum (FBS; Gibco, USA) and 1% Penicillin–Streptomycin (Gibco, USA). Cells were seeded at a density of 2 × 10^4^ cells per well and treated with Carrimycin or ISP I in the presence or absence of R406 (MedChemExpress, USA, Cat No. HY-12067) for 24–72 h. Cell viability was assessed using the Cell Counting Kit-8 (CCK-8; VitaScientific, USA) according to the manufacturer’s instructions. Combinational effects were evaluated using the SynergyFinder Highest Single Agent (HSA) model, with scores interpreted as follows: synergy, > 10; additive effect, − 10 to 10; antagonism, <  − 10 [22].

### EdU cell proliferation assay

Cell proliferation was assessed using an EdU incorporation assay kit (LifeSct, USA, Cat No. C0018). Following 24 h of Carrimycin (20 μM) treatment, cells were harvested, fixed with 3.7% paraformaldehyde, and permeabilized with 0.5% Triton X-100 (Sigma, USA). EdU incorporation was detected using LiFluor 594 azide according to the manufacturer’s instructions, and nuclei were counterstained with Hoechst 33,342. Images were captured using a Nikon C2 confocal microscope.

### Apoptosis and cell cycle analysis

Following 24 or 48 h of treatment with Carrimycin, alone or in combination with R406, apoptosis was assessed by flow cytometry using FITC-Annexin V and propidium iodide (PI) staining (BD Biosciences, CA, USA). To validate caspase dependency, rescue experiments were performed using the caspase inhibitor Z-VAD-FMK (MedChemExpress, USA, Cat No. HY-16658 B). For cell cycle analysis, Carrimycin-treated cells were fixed in 70% ethanol overnight at 4 °C, followed by staining with FxCycleTM PI/RNase Staining Solution (ThermoFisher Scientific, USA, Cat No. F10797), and analyzed by flow cytometry (BD Biosciences, CA, USA).

#### Western blot

Cell lysates were prepared in RIPA buffer (ThermoFisher Scientific, USA) supplemented with 1 mM PMSF and 1 × phosphatase inhibitor (Cell Signaling Technology, USA) and incubated on ice for 30 min. After quantification via a BCA assay, equal amounts of protein were mixed with 4 × loading buffer and boiled at 98 °C for 10 min. Protein samples were separated by electrophoresis using NuPAGE 4–12% Bis–Tris gels (ThermoFisher Scientific, USA) and transferred to nitrocellulose membranes. Membranes were blocked with 5% non-fat milk for 1 h at room temperature and then incubated overnight at 4 °C with primary antibodies. After washing three times with TBST, membranes were incubated with HRP-conjugated secondary antibodies for 1.5 h at room temperature. Protein bands were visualized using an enhanced chemiluminescence (ECL) detection kit (ThermoFisher Scientific, USA) and imaged with a ChemiDoc system (Bio-Rad, USA). Details of the antibodies used are provided in Table 1.

**Table 1:** xx

Primary antibody	Company	Concentration	Catalog number
Cleaved Caspase − 3	Cell Signaling Technology	1:1000	#9661
Cleaved PARP	Cell Signaling Technology	1:1000	#5625
Bcl-xL	Cell Signaling Technology	1:1000	#2764
Cyclin D3	Cell Signaling Technology	1:1000	#2936
CDK4	Cell Signaling Technology	1:1000	#12790
CDK6	Cell Signaling Technology	1:1000	#13331
Phospho-Src Family	Cell Signaling Technology	1:1000	#6943
Phospho-Syk	Cell Signaling Technology	1:1000	#2710
Phospho-CD79A	Cell Signaling Technology	1:1000	#5173
Syk	Cell Signaling Technology	1:2000	#13198
Phospho-Akt	Cell Signaling Technology	1:1000	#4060
Akt	Cell Signaling Technology	1:2000	#9272
TCF3	Santa Cruz Biotechnology	1:1000	sc-133075
beta-Actin	Cell Signaling Technology	1:5000	#3700
γ-H2AX	Cell Signaling Technology	1:1000	#2577

#### Intracellular reactive oxygen species (ROS) analysis

Intracellular ROS levels were quantified using the CellROX™ Green Reagent (ThermoFisher Scientific, USA, Cat No. C10444) according to the manufacturer’s instructions. Briefly, Raji and NAMALWA cells were treated with indicated concentrations of Carrimycin for 24 h. For rescue experiments, cells were co-treated with 10 mM N-acetylcysteine (NAC; MedChemExpress, USA, Cat No. HY-B0215) to scavenge excess ROS. Following treatment, cells were incubated with CellROX™ Green Reagent (final concentration, 5 µM) in complete medium at 37 °C in the dark for 30 min. Cells were then washed three times with phosphate-buffered saline (PBS) to remove excess dye. The fluorescence intensity was measured using flow cytometry (BD Biosciences, CA, USA) or C2 confocal microscopy (Nikon, USA).

#### Transcriptome sequencing

Differential gene expression between control and Carrimycin-treated groups (n = 3) was analyzed using the DESeq2 package, with significance defined as an adjusted* P*-value (*P*adj) ≤ 0.05 and |log2FoldChange| ≥ 1. For functional analysis, Gene Set Enrichment Analysis (GSEA) was performed utilizing the Gene Ontology Biological Process (GO BP) pathway sets. Enrichment scores were calculated using 1,000 permutations with a weighted statistic, with significance defined as *P* < 0.05 and false discovery rate (FDR) < 0.05.

#### Molecular docking and molecular dynamics (MD) simulation

Molecular docking was performed using the crystal structure of the TCF3 basic helix-loop-helix (bHLH) domain (RCSB PDB: 3U5V, covering ~ 65 residues). The predicted binding pocket maps to the core dimerization interface of the bHLH domain, a functionally critical region involved in subunit interaction and ID3 regulation. The 3D structure of ISP I (PubChem: 101,485,376) was docked into the active site of human TCF3 (RCSB PDB: 3U5V) using AutoDock Vina (v1.2.5) after standard ligand and receptor preparation in PyMOL and AutoDockTools.

Molecular dynamics (MD) simulations of the protein–ligand complexes were then conducted for 100 ns using GROMACS 2022.2. The system was modeled utilizing the Amber99SB-ILDN force field for the protein and GAFF2 parameters (sobtop_1.0) for the ligand. The complex was solvated in a TIP3P cubic box, neutralized with 0.15 M NaCl, energy-minimized, and equilibrated under NVT and NPT ensembles (300 K, 1 bar). Following complete equilibration, a 100 ns unconstrained MD production run was executed. Trajectory data were processed using standard GROMACS analysis tools. Structural stability and local residue flexibility were evaluated by calculating the backbone root-mean-square deviation (RMSD) and the root-mean-square fluctuation (RMSF), respectively. The protein's overall spatial compactness and protein-solvent interactions were assessed using the radius of gyration (Rg) and solvent-accessible surface area (SASA). Intermolecular stability was characterized by quantifying protein–ligand hydrogen bonds. Finally, principal component analysis (PCA), combined with the gmx sham tool, was utilized to construct a free energy landscape (FEL) to elucidate the system's major conformational states and energy distribution characteristics.

### Cellular thermal shift assay (CETSA)

Raji and NAMALWA cells were lysed via five rapid freeze–thaw cycles in liquid nitrogen and subsequently centrifuged at 12,000 × g for 20 min at 4 °C to isolate the soluble protein lysate. The lysate was incubated with Carrimycin (20 µM) or a DMSO vehicle control for 2 h. Following incubation, the samples were aliquoted into PCR tubes, heated across a temperature gradient for 3 min using a thermal cycler, and immediately cooled to 4 °C. The remaining soluble supernatants were then analyzed by Western blotting.

#### Cycloheximide (CHX) chase assay

To measure protein half-life, Raji and NAMALWA cells were treated with the protein synthesis inhibitor cycloheximide (CHX, 100 µg/mL; Sigma, USA) in the presence of DMSO or Carrimycin (20 µM) for 0, 0.5, 1, 2, 4, and 8 h. Total cell lysates were prepared in RIPA buffer and analyzed by Western blotting.

#### Cellular proteasome and lysosome inhibition assay

To determine whether Carrimycin-induced TCF3 degradation is mediated by the ubiquitin–proteasome system or the lysosome-autophagy pathway, Raji and NAMALWA cells were co-treated with Carrimycin (20 µM) in the presence of the proteasome inhibitor MG132 (5 µM; MedChemExpress, USA, Cat. No.: HY-13259) or the lysosome inhibitor Bafilomycin A1 (100 nΜ; MedChemExpress, USA, Cat. No.: HY-100558). Following a 6 h co-treatment, total cell lysates were harvested in RIPA buffer and subjected to Western blot analysis using an anti-TCF3 primary antibody.

#### Drug affinity responsive target stability (DARTS) assay

Raji and NAMALWA cells were washed twice with PBS and subsequently lysed using RIPA solution. Afterwards, the cell lysates were incubated with different concentrations of Carrimycin (0, 5, 10, and 20 µM) or vehicle control at room temperature for 1 h. Limited proteolysis was subsequently performed by adding Pronase E (MedChemExpress, USA, Cat No. HY-114158 A) at a 1:1000 mass ratio (wt/wt, Pronase-to-total protein) and digesting at room temperature for 30 min. The digestion was terminated immediately by adding 4 × SDS loading buffer and boiling samples at 95 °C for 10 min. Protein stability and enzymatic resistance were analyzed by Western blotting with specific antibodies against TCF3.

#### Lentiviral knockdown

Short hairpin RNA (shRNA) targeting human TCF3 was purchased from Santa Cruz Biotechnology (Dallas, TX, USA; Cat No. sc-63307-V). NAMALWA cells were transduced with lentiviral supernatants and selected with puromycin (2 μg/ml) to establish stable knockdown lines (NAMALWA-shTCF3), validated by Western blotting.

#### Immunohistochemistry (IHC)

Paraffin-embedded tissue sections were deparaffinized and rehydrated, followed by heat-induced antigen retrieval in either citrate buffer (pH 6.0) or Tris–EDTA (pH 9.0). After quenching endogenous peroxidase with 3% H_2_O_2_ and blocking non-specific binding with Background Punisher (BIOCARE, USA), sections were incubated with primary antibodies overnight at 4 °C. Signal detection was performed using the MACH4 HRP-Polymer (BIOCARE, USA), and visualized with DAB substrate (Vector Laboratories, USA), followed by hematoxylin counterstaining. Slides were subsequently dehydrated, cleared, and mounted with Permount (ThermoFisher Scientific, USA). Primary antibodies are listed in Table 2. IHC images were analyzed using R (v4.5.1) and EBImage (v4.52.0) [23]. Following scale-bar exclusion and hematoxylin–DAB color separation, candidate nuclei were segmented using local background correction and watershed segmentation. Mean DAB optical density was measured in nuclear regions for Ki67 and TCF3 and in approximate perinuclear regions for P-SYK. Objects were classified as negative, weak, moderate, or strong using thresholds of 0.10, 0.25, and 0.50, applied consistently within each marker. Positive staining was expressed as the percentage of scored objects above the positivity threshold. H-scores were calculated as (% weak) + 2 × (% moderate) + 3 × (% strong).

**Table 2:** xx

Primary antibody	Company	Concentration	Catalog number
Ki-67	ThermoFisher Scientific	1:200	MA5-14,520
TCF3	Santa Cruz Biotechnology	1:200	sc-133075
Phospho-Syk	Cell Signaling Technology	1:200	#2710

### Tumor models

Animal studies were approved by the National Cancer Institute (NCI) Animal Care and Use Committee (ACUC). Female NOD *scid* gamma (NSG) mice (6–8 weeks old) were obtained from the NCI CCR Research Model Systems Program.

For the subcutaneous tumor model, Raji cells were resuspended in PBS (5 × 10^6^ cells/100 μL) and injected subcutaneously into each mouse. When tumors reached a volume of 150–200 mm^3^ (approximately 10 days post-implantation), mice were randomized into two groups (n = 6 per group). Mice received daily intraperitoneal injections of vehicle or Carrimycin (55 mg/kg) for 8 consecutive days. Tumor size was measured every 2 days using calipers, and tumor volume was calculated using the formula: V = (length × width^2^)/2. At the end of the treatment period, mice were euthanized, and tumors were harvested for further analysis.

For the intracranial implantation model, mice were anesthetized and subjected to stereotactic intracranial injection. Luciferase-expressing Raji (Raji-luc) cells (1 × 10^5^ in 2 µL) were resuspended in Hank's Balanced Salt Solution (HBSS; Crystalgen) and injected into the right brain (1 mm rostral of the bregma, 2 mm right of midline, and 2 mm deep from the skull surface). Tumor growth was monitored every 3 days by bioluminescence imaging (BLI) using a Xenogen In Vivo Imaging System (IVIS). Seven days after implantation, mice were randomized into two groups based on bioluminescence signal intensity and treated daily with vehicle or Carrimycin (60 mg/kg). Body weight was recorded every two days. Mice were euthanized upon reaching survival endpoints, and tissues were collected for subsequent analysis.

### Statistical analysis

Statistical analyses were performed using GraphPad Prism software (version 10.6). Comparisons between two groups were analyzed using the two-tailed Student’s t-test, while comparisons among multiple groups were assessed using one-way ANOVA followed by Dunnett’s multiple comparisons test. Survival outcomes were estimated using the Log-rank (Mantel-Cox) test. Bioinformatics analyses were conducted using the R statistical computing environment (version 4.5). A *P*-value of < 0.05 was considered statistically significant. Data are shown as means ± SD of three independent experiments.

## Results

### Carrimycin exerts potent anti-proliferative effects on BL cells

**Fig. 1:**
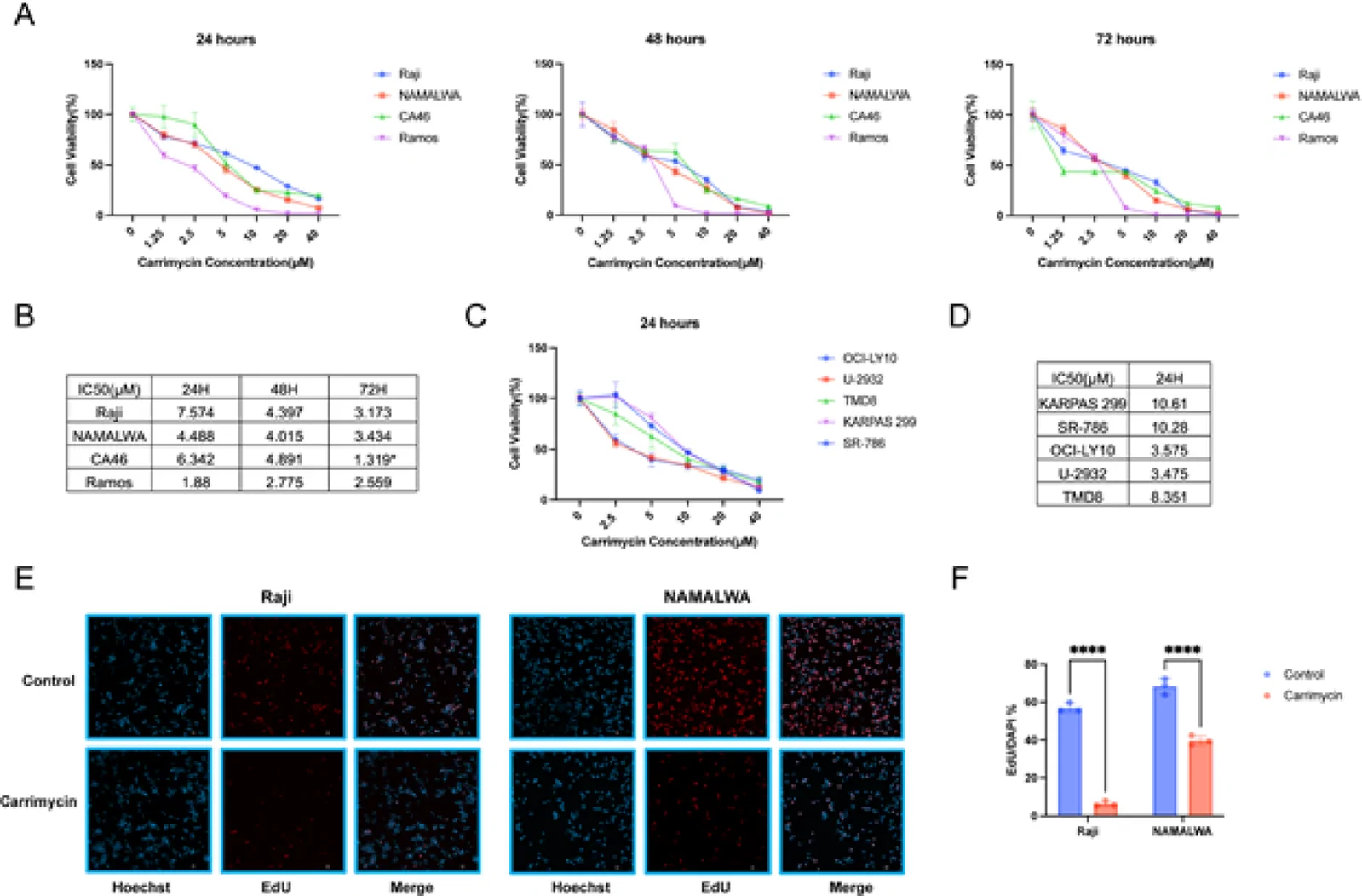
Carrimycin inhibits the proliferation of BL cell lines. **A** Cell viability of four BL cell lines (Raji, NAMALWA, CA46, Ramos) treated with indicated concentrations of Carrimycin at varying time points, assessed by CCK-8 assay. **B** The half-maximal inhibitory concentration (IC50) values of Carrimycin across the four BL cell lines. *Note: 4-parameter logistic (4PL) regression goodness-of-fit (R^2^) values across lines and time points ranged from 0.95 to 0.99, except for CA46 at 72 h (R^2^ = 0.8192). This lower R^2^ reflects a non-monotonic, biphasic profile with an intermediate plateau, and its IC50 primarily denotes sensitivity in the early transition phase. **C** CCK-8 viability assays evaluating Carrimycin cytotoxicity in two ALCL cell lines (KARPAS 299, SR-786) and three DLBCL cell lines (OCI-LY10, U-2932, TMD8). **D** IC50 values of Carrimycin across the five lymphoma cell lines at 24 h. **E**, **F** Representative images **E** and quantification **F** of EdU assays demonstrating reduced cellular proliferation in BL cell lines following 24 h of Carrimycin (20 μM) treatment. **** P < 0.0001

To evaluate the cytotoxic effects of Carrimycin, we performed CCK-8 assays in four BL cell lines (Raji, NAMALWA, CA46, and Ramos) as well as in cell lines representing other lymphoma subtypes. Carrimycin significantly suppressed cell proliferation in a dose-dependent manner in all BL cell lines at different time points (24, 48, and 72 h) (Fig. 1A). The half-maximal inhibitory concentration (IC50) at 24 h was 7.574 µM, 4.488 µM, 6.342 µM, and 1.88 µM for Raji, NAMALWA, CA46, and Ramos cells, respectively (Fig. 1B). Notably, at 72 h, CA46 exhibited a non-monotonic biphasic response (goodness-of-fit R^2^ = 0.8192), showing an early plateau followed by further suppression at higher concentrations, indicating that its calculated IC50 (1.319 µM) predominantly reflects the initial sensitivity window. Consistently, Carrimycin also exhibited broad anti-proliferative activity across additional lymphoma subtypes (Fig. 1C and D), supporting its potential as a therapeutic agent in lymphoma. Carrimycin is composed of three main components (ISP I, II, and III), of which ISP I represents the primary active component [21]. Accordingly, purified ISP I exerted potent inhibitory effects across all four BL cell lines (Supplementary Fig. 1A and 1B). Based on these profiles, Raji and NAMALWA cells were selected for subsequent mechanistic studies. Further validation using EdU assays demonstrated a significant reduction in DNA synthesis following 24 h of Carrimycin treatment (Fig. 1E and F). Collectively, these data demonstrate that Carrimycin exerts potent anti-proliferative effects across multiple BL cell lines.

### Carrimycin induces apoptosis and arrests the cell cycle at the G0/G1 phase in BL cells

**Fig. 2:**
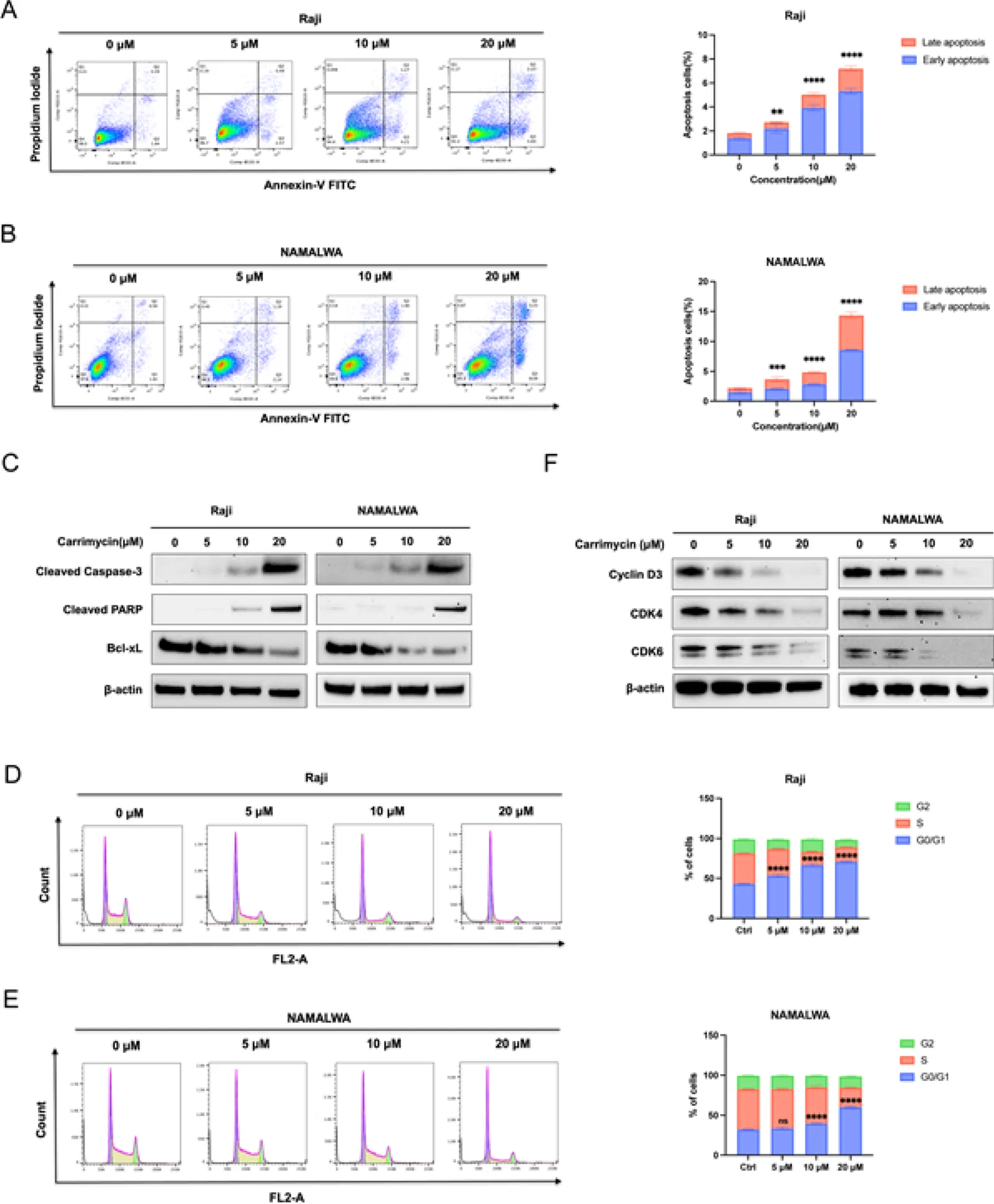
Carrimycin triggers G0/G1 cell cycle arrest and apoptosis in BL cells. **A**, **B** Representative flow cytometry plots and quantitative analysis of apoptosis (Annexin V/PI staining) in Raji **A** and NAMALWA (B) cells treated with gradient concentrations of Carrimycin (5, 10, and 20 μM) for 24 h. Representative flow cytometry plots are shown on the left, and the corresponding quantitative analysis is shown on the right. **C** Western blot analysis of apoptosis-related proteins (cleaved PARP, cleaved Caspase − 3, and Bcl-xL) in Carrimycin-treated BL cells. **D**, **E** Cell cycle distribution analyzed by flow cytometry in Raji **D** and NAMALWA **E** cells treated with Carrimycin. Representative flow cytometry plots are displayed on the left, with the corresponding quantitative analysis on the right. **F** Western blot analysis of cell cycle regulatory proteins Cyclin D3 and CDK4/6 following Carrimycin treatment for 24 h. **P < 0.01, ***P < 0.001, **** P < 0.0001

To determine whether Carrimycin-induced cytotoxicity was driven by apoptosis, we performed Annexin V/PI flow cytometry. Treatment with Carrimycin (5, 10, and 20 µM) for 24 h resulted in a dose-dependent increase in both early (Annexin V+/PI- cells) and late (Annexin V+/PI + cells) apoptotic populations in Raji (Fig. 2A) and NAMALWA cells (Fig. 2B). Western blot analysis revealed that Carrimycin treatment upregulated the pro-apoptotic markers cleaved PARP and cleaved Caspase − 3, while downregulating the anti-apoptotic protein Bcl-xL (Fig. 2C). To confirm the involvement of caspase-dependent pathways, we performed rescue experiments using the pan-caspase inhibitor Z-VAD-FMK (100 µM). Co-treatment with Z-VAD-FMK significantly attenuated Carrimycin-induced apoptosis (Supplementary Fig. 2A and 2B). Consistently, these data indicate that Carrimycin triggers caspase-dependent apoptosis in BL cells.

To further elucidate the mechanism of growth inhibition, we analyzed the cell cycle distribution. Treatment with increasing concentrations of Carrimycin (5, 10, and 20 µM) resulted in significant cell cycle arrest at the G0/G1 phase in both Raji and NAMALWA cells (Fig. 2D and E). Given that the G1-S phase transition in BL is critically regulated by Cyclin D3 and its kinase partners CDK4/6, and that CCND3 mutations drive proliferation in ~ 38% of sporadic BL cases [16], we examined the impact of Carrimycin on this pathway. Western blotting revealed a dose-dependent downregulation of Cyclin D3 and CDK4/6 protein levels (Fig. 2F). Taken together, these findings show that Carrimycin induces G0/G1 arrest by disrupting the CDK4/6-Cyclin D3 axis.

In addition, to explore the temporal relationship between growth inhibition and cell death, BL cells were evaluated at an extended 48-h time point. Flow cytometric analysis revealed a dose-dependent surge in Annexin-V + apoptotic cells, reaching ~ 57% in Raji and ~ 38% in NAMALWA cells at 20 µM (Supplementary Fig. 2C–D). These observations indicate that while the acute (24 h) reduction in cell viability is largely driven by G0/G1 arrest, sustained Carrimycin exposure culminates in extensive apoptotic clearance.

### Carrimycin induces DNA damage in BL cells via ROS accumulation.

**Fig. 3:**
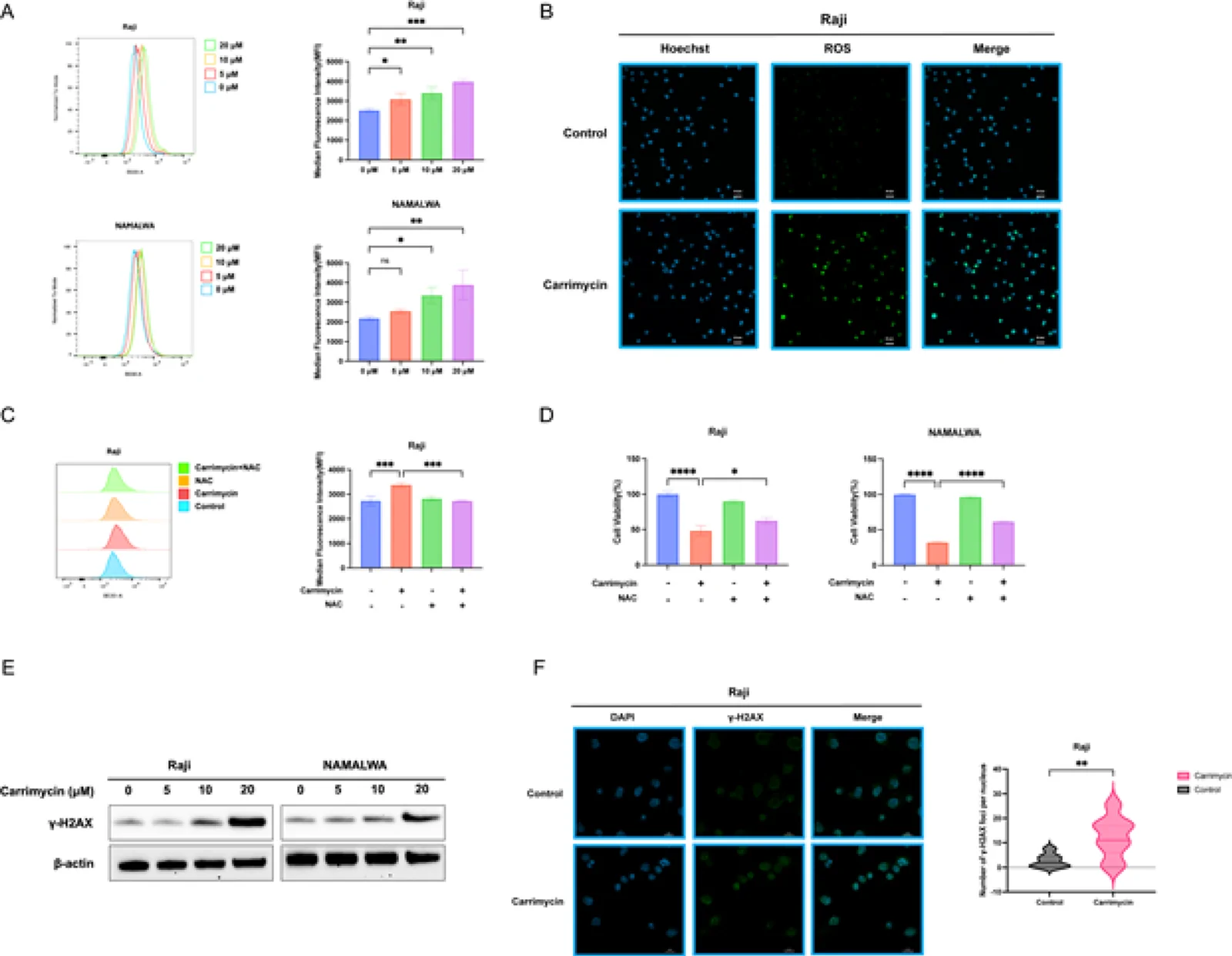
Carrimycin drives DNA damage in BL cells by promoting the accumulation of ROS **A** Flow cytometric analysis of intracellular reactive oxygen species (ROS) levels in Raji (upper) and NAMALWA (lower) cells using the CellROX Green probe following dose-dependent Carrimycin treatment. The left panel shows representative flow cytometry plots, and the right panel shows quantitative analysis. **B** Representative fluorescence microscopy images of ROS in Carrimycin-treated Raji cells. **C** Flow cytometric analysis of Carrimycin-induced (10 μM) ROS levels in Raji cells by the addition of 10 mM NAC. The left are representative flow cytometry plots, and the right is quantitative analysis. **D** Cell viability upon co-treatment with Carrimycin (10 μM) and NAC (10 mM) in Raji and NAMALWA cells for 24 h. **E** Western blot analysis of the expression of γ-H2AX in Raji and NAMALWA cells following carrimycin treatment. **F** Representative confocal immunofluorescence images (left) and quantitative analysis of the number of nuclear γ-H2AX foci per cell (right) in Raji cells treated with vehicle or Carrimycin (20 µM) for 24 h. *P < 0.05, **P < 0.01, ***P < 0.001, **** *P* < 0.0001.

Previous studies have characterized Carrimycin as a pro-oxidant that exerts anti-tumor effects via oxidative stress [21, 24]. Consistent with this, staining with the CellROX Green probe revealed a dose-dependent accumulation of ROS in Carrimycin-treated Raji and NAMALWA cells through flow cytometry and immunofluorescence (Fig. 3A, B, and Supplementary Fig. 3A). To confirm the functional role of ROS, we performed rescue experiments using the ROS scavenger N-acetylcysteine (NAC, 10 mM). NAC treatment effectively blocked ROS accumulation (Fig. 3C and Supplementary Fig. 3B) and significantly attenuated Carrimycin-induced cytotoxicity (Fig. 3D). Because excessive ROS accumulation is a recognized driver of DNA damage in tumor cells[25], we evaluated the expression of the DNA damage marker γ-H2AX. Both Western blotting and immunofluorescence analyses revealed that Carrimycin treatment markedly upregulates γ-H2AX levels, confirming an induction of DNA damage in both cell lines (Fig. 3E, F, and Supplementary Fig. 3C). In conclusion, these findings demonstrate that Carrimycin-induced ROS accumulation drives DNA damage.

### Carrimycin suppresses B-cell receptor (BCR) signaling in BL cells

**Fig. 4:**
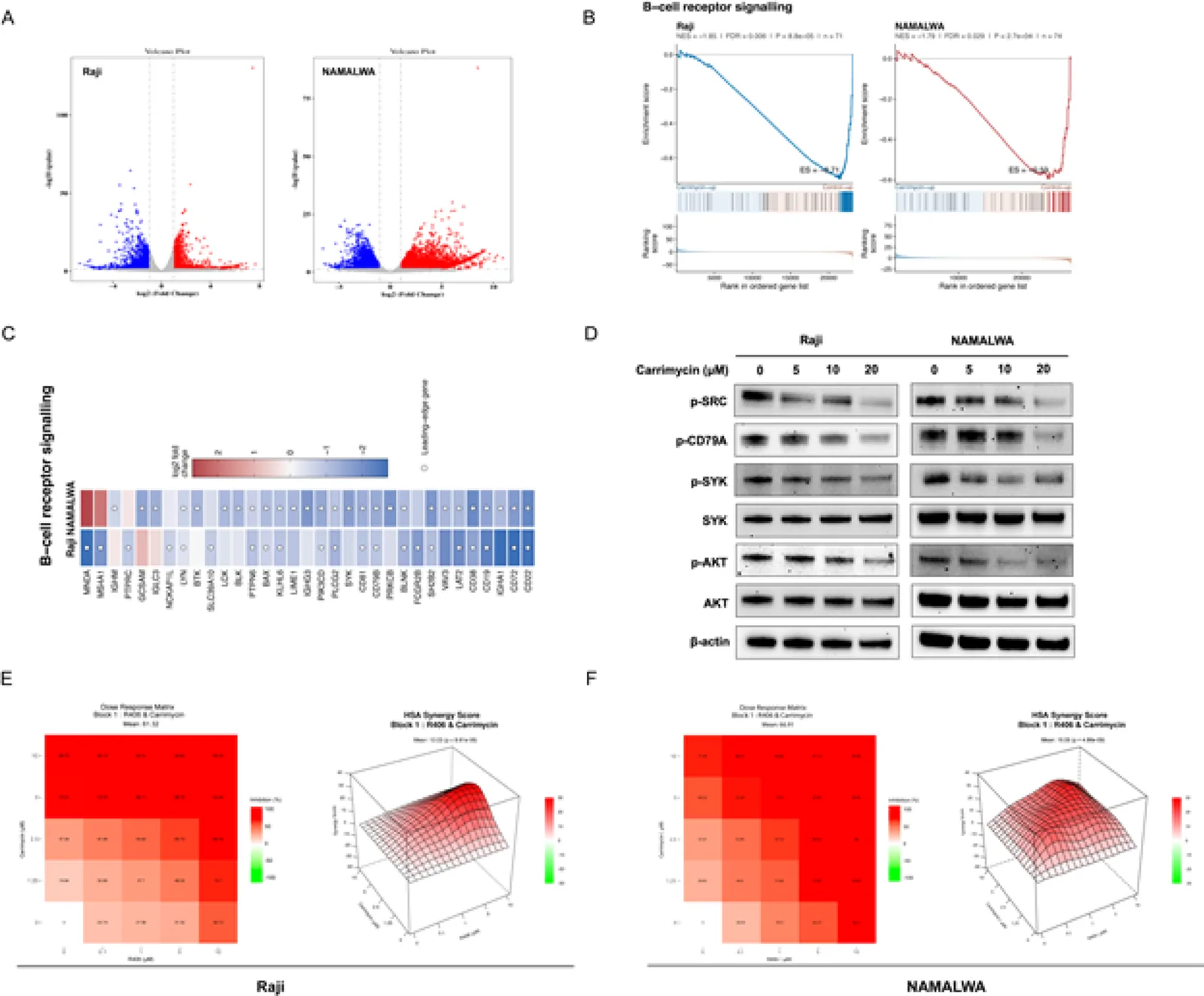
Carrimycin downregulates tonic BCR signaling. **A** Volcano plots illustrating differentially expressed genes (DEGs) in Raji and NAMALWA cells following Carrimycin treatment for 24 h. **B** Gene Set Enrichment Analysis (GSEA) plots for the BCR signaling pathway in Raji and NAMALWA cells. **C** Heatmap depicting the log_2_ (Fold Change) of the core enriched genes within the BCR signaling pathway for both cell lines. **D** Western blot analysis evaluating the phosphorylation status of key BCR signaling effectors (p-CD79A, p-SYK, p-SRC) and the downstream survival target p-AKT after 24 h of Carrimycin exposure. **E** and **F** HSA synergy scores calculated via SynergyFinder for the combination of Carrimycin and the SYK inhibitor R406 in Raji cells **E** and NAMALWA cells **F**

To further elucidate the molecular mechanism of Carrimycin in BL, we performed transcriptome RNA sequencing on Raji and NAMALWA cells following treatment. Transcriptomic profiling revealed widespread gene expression alterations in both cell lines, as visualized by volcano plots (Fig. 4A). Gene Set Enrichment Analysis (GSEA) demonstrated a negative enrichment score for BCR signaling in both Raji and NAMALWA cells, indicating that Carrimycin downregulates this pathway (Fig. 4B). A heatmap of the core-enriched genes in this set further highlighted the consistent transcriptional repression of BCR signaling components across both cell lines (Fig. 4C).

Then, we validated these findings via Western blot, focusing on key BCR effectors. Carrimycin treatment reduced the phosphorylation levels of CD79A, SYK, and SRC (Fig. 4D). In BL, tonic BCR signaling promotes survival primarily through the PI3K-AKT axis [10]. Consistent with this mechanism, we observed a downregulation of phosphorylated AKT (p-AKT) following treatment (Fig. 4D). To exploit BCR dependency, we evaluated the synergistic effects of Carrimycin in combination with the SYK inhibitor R406. Using the SynergyFinder HSA model, synergy scores of 13.03 and 15.05 were obtained for Raji and NAMALWA cells, respectively, indicating a synergistic interaction (Fig. 4E and F). Time-course analysis further confirmed that this combination significantly suppressed growth at different time points (24, 48, and 72 h) (Supplementary Fig. 4A and 4B). Furthermore, flow cytometry demonstrated that the combination therapy significantly increased the rate of apoptosis compared to single-agent treatment (Supplementary Fig. 4C and 4D). In summary, these data suggest that Carrimycin induces cell death by suppressing the tonic BCR signaling axis.

### Carrimycin targets TCF3 in BL cells

**Fig. 5:**
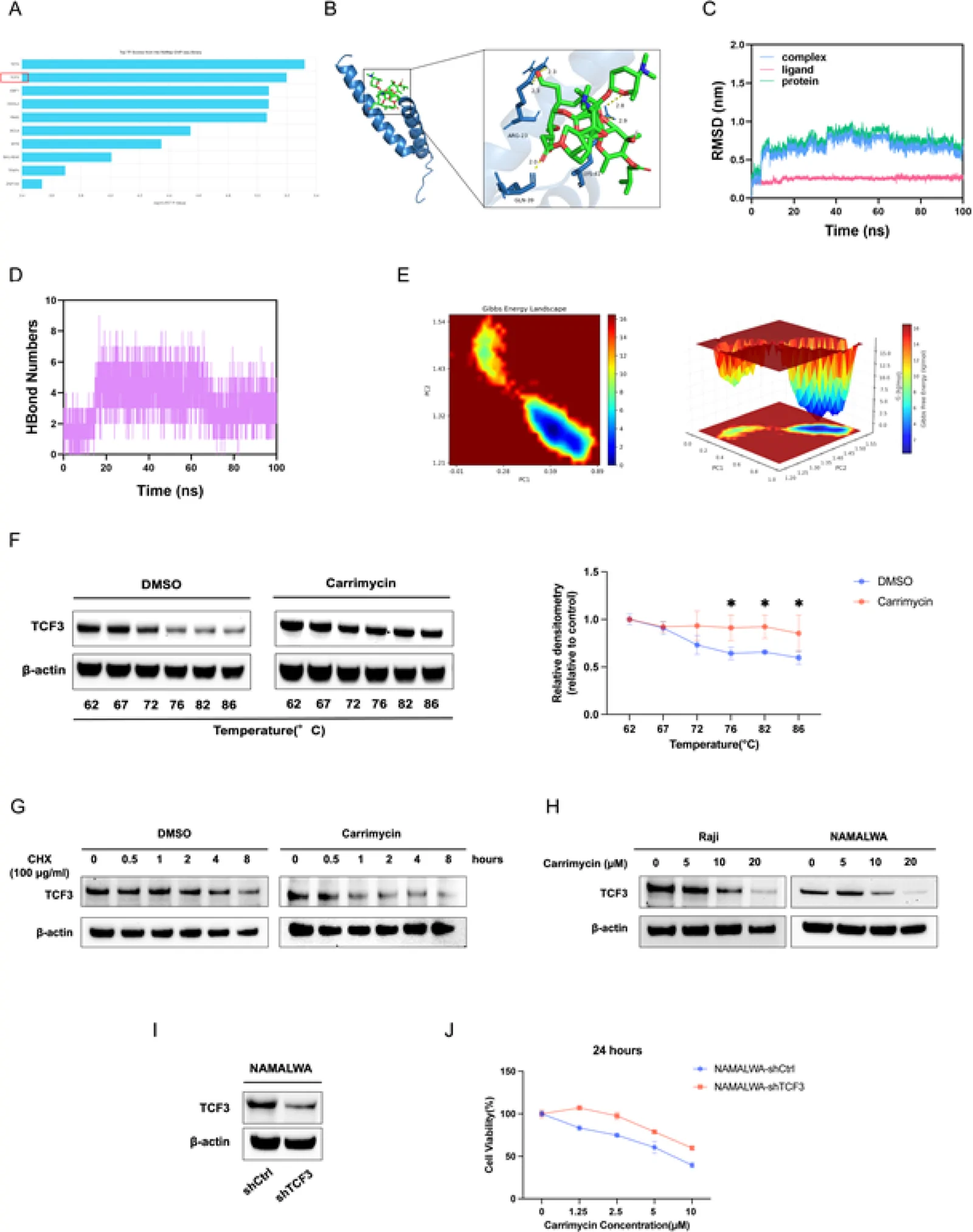
Carrimycin binds TCF3 to exert cytotoxic effects in BL cells. **A** Bioinformatic identification of candidate upstream transcription factors regulating shared DEGs in Carrimycin-treated cells using the ChEA3 platform integrated with ReMap ChIP-seq datasets. The red box highlights TCF3. **B** Computational molecular docking of ISP I into the active site of TCF3 (Binding Energy =  − 5.9 kcal/mol). **C**–**E** 100 ns molecular dynamics (MD) simulation validating the stability of the TCF3-ISP I complex, showing **C** root mean square deviation (RMSD) trajectories, **D** intermolecular hydrogen bond frequencies, and (E) the Free Energy Landscape (FEL). **F** Cellular Thermal Shift Assay (CETSA) in Raji cells treated with DMSO or Carrimycin across a 62–86 °C temperature gradient. Line graph quantification (right) demonstrates thermal stabilization of TCF3 by Carrimycin. **G** Cycloheximide (CHX, 100 µg/mL) chase assay in Raji cells co-treated with DMSO or Carrimycin for 0 to 8 h. (H) Western blot analysis of TCF3 protein expression in Raji and NAMALWA cells treated with increasing concentrations of Carrimycin (0, 5, 10, 20 µM) for 24 h. **I** Western blot validation of stable shRNA-mediated knockdown of TCF3 in NAMALWA cells. **J** CCK-8 cell viability curves of NAMALWA-shCtrl and NAMALWA-shTCF3 cells treated with graded concentrations of Carrimycin (0–10 µM) for 24 h

To identify the upstream molecular drivers responsible for Carrimycin-altered BCR signaling, we analyzed the shared DEGs from sequence data using the ChEA3 transcription factor prediction platform [26]. Integration of these results with ReMap ChIP-seq datasets identified the transcription factor TCF3 as a primary candidate. (Fig. 5A). Given that TCF3 is a well-documented master regulator of BCR signaling in Burkitt lymphoma [16], we hypothesized that Carrimycin targets this transcription factor to abrogate BCR signaling. To evaluate whether Carrimycin targets TCF3, we performed computational molecular docking. Because Carrimycin is a multicomponent antibiotic complex, we modeled the interaction using its principal bioactive monomer, ISP I. Docking simulations revealed that ISP I favorably binds the active pocket of TCF3 with a binding affinity of − 5.9 kcal/mol, forming stable hydrogen bonds with LYS41, ARG23, and GLN39 (Fig. 5B and Supplementary Fig. 5A). A subsequent 100 ns molecular dynamics (MD) simulation confirmed the dynamic stability of the TCF3-ISP I complex. The system rapidly reached equilibrium, exhibiting minimal root-mean-square deviation (RMSD) fluctuations (Fig. 5C), an average of four persistent intermolecular hydrogen bonds (Fig. 5D), and a well-defined global free energy minimum (Fig. 5E). Further trajectory analyses demonstrated sustained structural compactness (radius of gyration, Rg) and consistent solvent exposure (solvent-accessible surface area, SASA) (Supplementary Fig. 5B and 5C). Additionally, low root-mean-square fluctuation (RMSF) values indicated restricted residue flexibility within the binding pocket throughout the simulation (Supplementary Fig. 5D). Collectively, these metrics support a stable ligand-receptor interaction.

To validate this physical interaction, we performed a cellular thermal shift assay (CETSA). Carrimycin treatment significantly enhanced the thermal stability of TCF3 in Raji and NAMALWA cells at higher temperatures compared to the vehicle control (Fig. 5F and Supplementary Fig. 5E). To further corroborate these findings, a Drug Affinity Responsive Target Stability (DARTS) assay was conducted using cell lysates. Escalating concentrations of Carrimycin progressively protected TCF3 from Pronase E-mediated proteolysis in a dose-dependent manner (Supplementary Fig. 5F).

To determine whether this reduction was due to altered protein stability, we conducted a cycloheximide (CHX) chase assay. Carrimycin shortened the half-life of TCF3 compared to the DMSO control (Fig. 5G and Supplementary Fig. 5G). To define the specific degradation machinery responsible for Carrimycin-accelerated TCF3 clearance, BL cells were co-treated with Carrimycin in the presence of either the proteasome inhibitor MG132 or the lysosomal inhibitor Bafilomycin A1. Western blotting revealed that MG132 treatment restored TCF3 protein abundance in both Raji and NAMALWA cells (Supplementary Fig. 5H), whereas Bafilomycin A1 failed to rescue TCF3 degradation (Supplementary Fig. 5I).

Next, we investigated the impact of Carrimycin on TCF3 protein expression. Western blot analysis revealed that Carrimycin treatment downregulates TCF3 levels in a dose-dependent manner (Fig. 5H). Finally, to establish whether TCF3 mediates the anti-tumor efficacy of Carrimycin, we generated stable TCF3-knockdown (shTCF3) NAMALWA cell lines (Fig. 5I). CCK-8 viability assays demonstrated that depleting TCF3 partially rescued NAMALWA cells from Carrimycin-induced cytotoxicity compared to control cells (Fig. 5J). To conclude, these data indicate that Carrimycin exerts its anti-lymphoma effects, at least in part, by binding TCF3.

### Carrimycin exhibits anti-tumor efficacy in subcutaneous and intracranial xenograft models

**Fig. 6:**
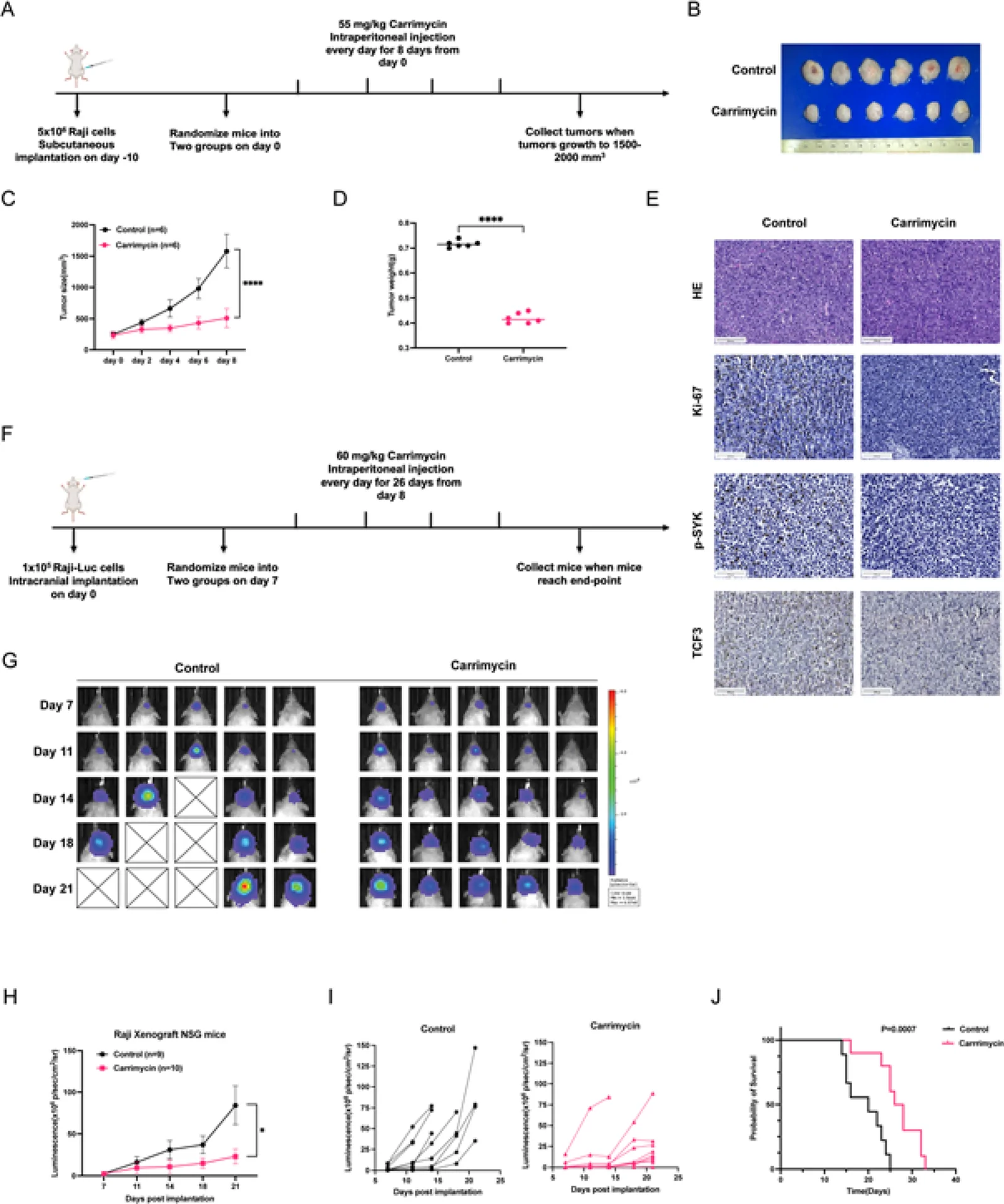
Carrimycin suppresses BL growth in subcutaneous and intracranial xenograft models. **A** Experimental schematic for the subcutaneous Raji xenograft model in NSG mice treated with Carrimycin (55 mg/kg via intraperitoneal injections) or vehicle control. (B-D) Quantification of tumor size **B**, **C** and final tumor weight **D** from subcutaneous xenografts showing growth suppression by Carrimycin. **E** Representative Hematoxylin and Eosin (H&E) and immunohistochemical (IHC) staining of subcutaneous tumor tissues for proliferation (Ki-67), BCR signaling (p-SYK), and TCF3. **F** Experimental design for the intracranial tumor model established via injection of luciferase-expressing Raji cells. **G** Representative bioluminescence images monitoring intracranial tumor growth in mice from the vehicle and treatment groups. **H** Quantification of tumor bioluminescence intensity at 7, 11, 14, 18, and 21 days post-implantation. (I) Individual tumor growth trajectories for each mouse bearing intracranial xenografts in the vehicle control (Left) and Carrimycin-treated (Right) groups. (J) Kaplan–Meier survival curve demonstrating extended median overall survival (mOS) in the Carrimycin-treated intracranial cohort (27 days vs 20 days). *P < 0.05, **** P < 0.0001

To validate the therapeutic efficacy of Carrimycin in vivo, we established a subcutaneous xenograft model using Raji cells in NSG mice. When the tumor volume reached approximately 150–200 mm^3^, mice were randomized and treated with Carrimycin (55 mg/kg, intraperitoneal injections) or vehicle control (Fig. 6A). Carrimycin treatment significantly suppressed tumor progression, resulting in a reduction in both tumor size and weight compared to the control group (Fig. 6B, C, and D). IHC analysis of harvested tissues confirmed the mechanism of action in vivo: Carrimycin-treated tumors exhibited reduced Ki-67 expression and downregulation of p-SYK and TCF3 (Fig. 6E and Supplementary Fig. 6D). Given the clinical challenge of treating BL with CNS involvement, we further evaluated Carrimycin in an orthotopic intracranial model (Fig. 6F). Luciferase-expressing Raji cells were implanted into the right brain, and tumor burden was monitored via bioluminescent imaging. Carrimycin treatment significantly suppressed intracranial tumor progression (Fig. 6G, H, and I) and extended median overall survival (mOS) from 20 days in the control group to 27 days in the treatment cohort (Fig. 6J). Body weight was monitored regularly to evaluate systemic tolerability for 10 days (Supplementary Fig. 6A). Mice treated with Carrimycin maintained stable body weights throughout the 10-day observation window. In addition, H&E staining of major organs revealed no significant pathological toxicity (Supplementary Fig. 6B). Consistent with the subcutaneous model, IHC analysis of intracranial tumors confirmed the suppression of Ki-67, p-SYK, and TCF3 (Supplementary Fig. 6C and 6D). Together, these data demonstrate that Carrimycin exerts anti-tumor activity in both subcutaneous and intracranial BL models.

## Discussion

While traditionally recognized for inhibiting bacterial protein synthesis, macrolide antibiotics are increasingly being investigated for their antineoplastic properties [27]. Carrimycin, a recombinant DNA-engineered macrolide derivative, has demonstrated cytotoxic activity across multiple malignancies [19–21, 28]. In the present study, we establish Carrimycin as a potent therapeutic agent against Burkitt lymphoma (BL) and delineate its dual mechanism of action: driving oxidative stress and dismantling the TCF3-BCR survival axis.

Carrimycin suppressed BL cell proliferation in a dose-dependent manner and markedly reduced DNA synthesis, as evidenced by EdU incorporation. Mechanistically, Carrimycin induced caspase-dependent apoptosis, characterized by the cleavage of caspase − 3 and PARP and the downregulation of the anti-apoptotic protein Bcl-xL. The reversal of these effects by Z-VAD-FMK further confirmed this apoptotic dependency. In parallel, Carrimycin exerted its anti-tumor effects by disrupting cell-cycle progression. Flow cytometric analysis revealed a dose-dependent G0/G1 phase arrest in Carrimycin-treated BL cell lines. This is highly clinically relevant in BL, where *CCND3* mutations frequently stabilize Cyclin D3 isoforms that, in complex with CDK4/6, aberrantly force the G1-S transition [29]. Carrimycin reduced Cyclin D3 and CDK4/6 expression, effectively destroying this oncogenic cell-cycle machinery. Of note, the kinetics of Carrimycin cytotoxicity in BL involve an immediate cytostatic shutdown followed by cumulative apoptosis. At 24 h, the marked suppression of metabolic viability in CCK-8 assays is primarily underpinned by G0/G1 cell cycle arrest, while apoptotic execution escalates significantly over 48 h.

Building on our previous findings that ISP I (the primary active component of Carrimycin) induces fatal ROS accumulation in solid tumors [24], we confirmed that oxidative stress is an important driver of its anti-lymphoma activity. CellROX Green staining revealed ROS accumulation following Carrimycin treatment, which was abrogated by co-treatment with the ROS scavenger N-acetylcysteine (NAC), concomitant with restoration of cell viability. Pathological ROS elevation is known to disrupt intrinsic hydrogen bonding, precipitating the unfolding and widespread fragmentation of the DNA double helix [30]. Indeed, exploiting ROS-mediated DNA damage represents a well-documented therapeutic vulnerability in various lymphoid malignancies [31, 32]. Aligning with this paradigm, our analyses confirmed a Carrimycin-induced upregulation of the DNA damage marker γ-H2AX, indicating that the drug leverages unchecked oxidative stress to inflict DNA damage in BL cells.

**Fig. 7:**
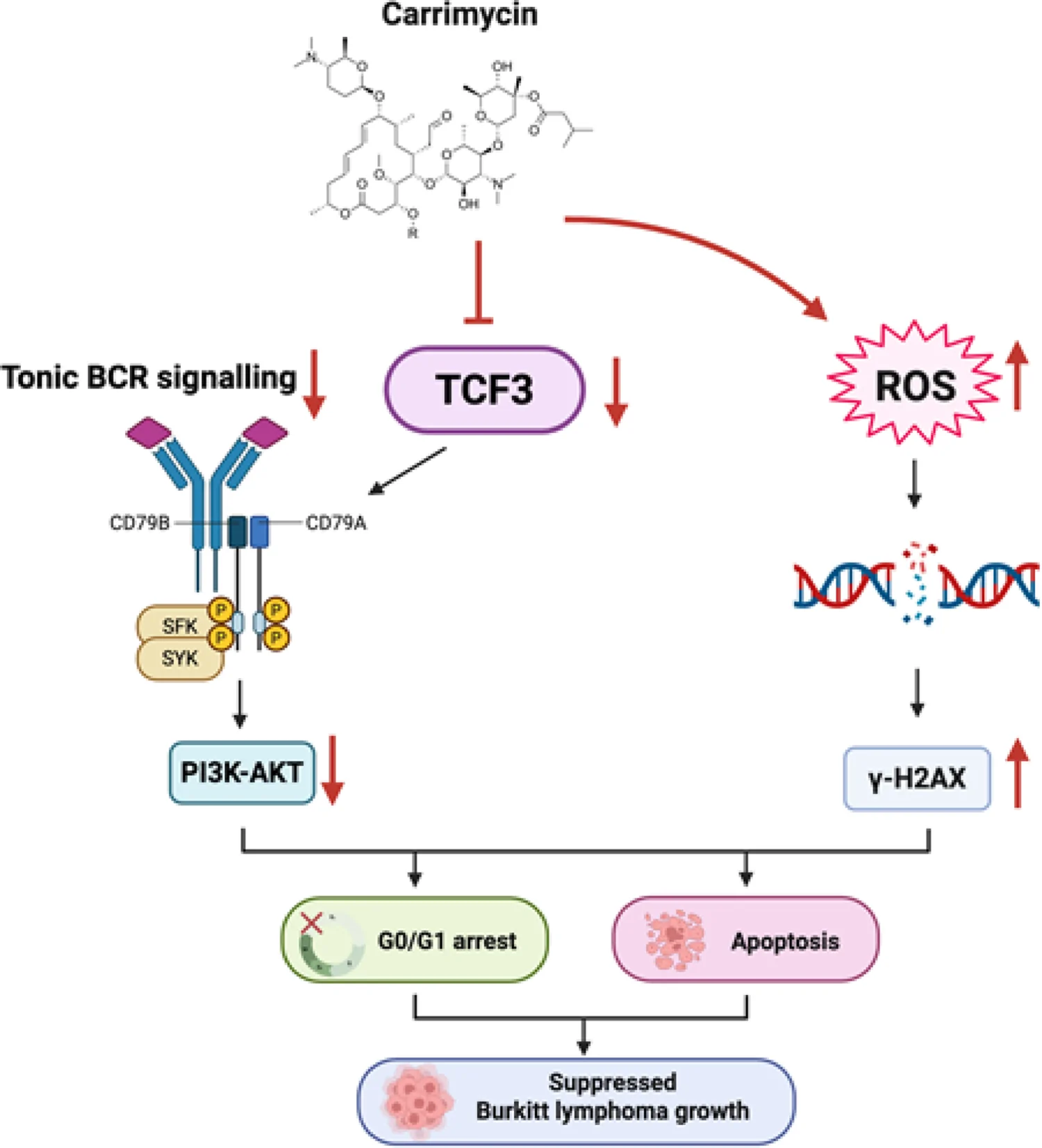
Schematic illustration of the proposed therapeutic mechanism of Carrimycin in Burkitt lymphoma. Carrimycin suppresses Burkitt lymphoma through a dual mechanism of action. It promotes a reduction in TCF3 protein abundance and is associated with decreased TCF3 stability, inhibiting tonic B-cell receptor (BCR) signaling and its downstream PI3K-AKT survival axis. Simultaneously, Carrimycin triggers accumulation of reactive oxygen species (ROS), inducing DNA damage marked by elevated γ-H2AX levels. This convergence of BCR signal termination and oxidative stress drives G0/G1 cell cycle arrest and caspase-dependent apoptosis, thereby potentially halting tumor growth

To resolve the broader molecular perturbations induced by Carrimycin, transcriptomic profiling was performed, revealing a suppression of the BCR signaling pathway. In BL, antigen-independent tonic BCR signaling propagates primarily via the PI3K-AKT-mTOR axis, serving as an obligate lifeline for tumor survival [10, 33]. Genetic silencing of CD79A or SYK, which are important proximal components of BCR signaling, induces apoptosis in BL, underscoring the pathway’s oncogenic dependence [11, 34]. Consistent with this, Western blotting analysis demonstrated a reduction in the phosphorylation of CD79A, SYK, SRC, and AKT following 24 h of Carrimycin exposure, indicating suppression of proximal and downstream BCR signaling. Furthermore, co-treatment with the SYK inhibitor R406 produced synergistic cytotoxicity, further supporting the conclusion that Carrimycin targets tonic BCR signaling as a core mechanism of action (Fig. 7).

In dissecting the upstream regulation of this BCR collapse, our multi-omic integration identified the transcription factor TCF3 as the molecular target. In the pathogenesis of BL, mutations disrupting the TCF3-ID3 axis drive constitutive TCF3 activation. This unrestrained TCF3 activity synergizes with c-MYC to ensure tumor survival and proliferation by amplifying tonic BCR/PI3K signaling and transactivating *CCND3 *[35]. We established the physical basis for this disruption through molecular docking and dynamic simulations, which demonstrated that ISP I, the principal bioactive component of Carrimycin, forms a highly stable complex with the TCF3 protein. To substantiate cellular target engagement, we performed the cellular thermal shift assay (CETSA) and the Drug Affinity Responsive Target Stability (DARTS) assay, which respectively confirmed that Carrimycin confers thermal stabilization and resistance to proteolysis to TCF3. Notably, this binding functionally destabilizes the protein in cells, leading to a shortened TCF3 half-life and accelerated degradation. Consistent with this degradation paradigm, pharmacological inhibition of the 26 S proteasome with MG132 reversed Carrimycin-triggered TCF3 depletion in both BL lines, whereas lysosomal inhibition with Bafilomycin A1 failed to restore TCF3 levels, demonstrating that Carrimycin promotes TCF3 degradation selectively through the ubiquitin–proteasome pathway. The requirement of this mechanism was confirmed by our genetic knockdown studies, where silencing TCF3 (shTCF3) markedly attenuated the therapeutic efficacy of Carrimycin. Collectively, these findings indicate that Carrimycin reduces TCF3 protein abundance and apparent stability, destroying the BCR signaling network in BL.

We examined whether baseline genetic background was associated with Carrimycin sensitivity across our Burkitt lymphoma cell-line panel. CA46 and NAMALWA harbor ID3 mutations, whereas Raji and Ramos retain wild-type ID3 [36]. In contrast, TCF3 mutations have not been documented in CA46, Raji, or Ramos, and NAMALWA has been reported to retain wild-type TCF3 [16]. Despite these differences in genetic background, Carrimycin exhibited potent cytotoxicity across all four cell lines, with no strict relationship between drug sensitivity and ID3 mutational status. These findings suggest that Carrimycin activity is not restricted to an ID3-mutant genotype. Mechanistically, because Carrimycin engages TCF3 and promotes its degradation, it may suppress downstream tonic BCR/PI3K survival signaling regardless of whether TCF3 activity is driven by upstream ID3 loss or other lineage-associated mechanisms.

Finally, we validated the anti-tumor efficacy of Carrimycin using a subcutaneous xenograft model. Consistent with our in vitro findings, Carrimycin treatment significantly suppressed tumor growth and downregulated Ki67, TCF3, and a key BCR signaling marker (p-SYK), confirming that it targets the TCF3-BCR axis in vivo. Central nervous system (CNS) involvement remains a critical barrier to survival in BL. A recent multicenter study by Zayac et al. reported that patients with CNS disease have a 3-year overall survival of only 49%, compared to 74% in those without [8]. Moreover, standard intrathecal therapies are often insufficient due to limited penetration into the brain parenchyma and challenges with clinical implementation [4]. To address this unmet need, we evaluated Carrimycin in an orthotopic intracranial model. Carrimycin significantly delayed tumor progression and prolonged median survival (27 days vs. 20 days in controls). Importantly, analysis of body weight and vital organs revealed a favorable safety profile with minimal systemic toxicity. Collectively, these findings demonstrate in vivo efficacy of Carrimycin in both subcutaneous and intracranial BL models and suggest its potential as a therapeutic drug for high-risk and CNS-involved disease.

While our findings establish antineoplastic efficacy and a favorable therapeutic window across Burkitt lymphoma models, several study limitations should be noted. First, cytotoxicity was not evaluated in non-malignant B-cell or hematopoietic controls to establish an in vitro therapeutic index. Second, our in vivo efficacy serves as a proof-of-concept using a single dose without standard chemotherapy comparators; future studies should include dose escalation and head-to-head regimens. Third, while CETSA and DARTS confirm intracellular engagement of TCF3, cell-free biophysical validation (e.g., Surface Plasmon Resonance or Isothermal Titration Calorimetry) with recombinant proteins remains to be performed. Finally, given the structural homology within the class I bHLH family, paralog-wide selectivity profiling (such as against TCF4 and TCF12) will be required to define E-protein binding dynamics.

## Conclusion

In summary, Carrimycin emerges as a highly effective, pleiotropic therapeutic agent against Burkitt lymphoma (BL). Mechanistically, Carrimycin binds and degrades the transcription factor TCF3, thereby dismantling the tonic BCR signaling axis. This targeted disruption, combined with the induction of oxidative stress and DNA damage, drives G0/G1 cell cycle arrest and caspase-dependent apoptosis (Fig. 7). Importantly, this mechanism translates to in vivo anti-tumor activity. Carrimycin suppresses tumor burden and extends survival in both subcutaneous and orthotopic CNS xenograft models while maintaining a favorable toxicity profile. Collectively, its demonstrated systemic and intracranial efficacy positions Carrimycin as a compelling candidate for translational investigation, specifically addressing the critical unmet clinical need in refractory or CNS-involved BL.

## Supplementary Information

Below is the link to the electronic supplementary material.

Supplementary Material 1

## Data Availability

The data that support the findings of this study are available from the corresponding author upon reasonable request. The raw sequencing data supporting the findings of this study have been deposited in the NCBI Sequence Read Archive (SRA) under BioProject accession number PRJNA1535052.
