# Supplementary material for: Carrimycin suppresses the TCF3-BCR signaling axis and induces oxidative DNA damage in Burkitt lymphoma
Source: Apoptosis. 2026 Nov 11;31(12):263. doi: 10.1007/s10495-026-02477-x (PMC13754957; doi:10.1007/s10495-026-02477-x)
Supplement: Supplementary file 1 [file 10495_2026_2477_MOESM1_ESM.docx]

**Supplementary materials**

**Supplementary Figure 1.** (A) Viability of four BL cell lines (Raji, NAMALWA, CA46, Ramos) treated with ISP I, assessed via CCK-8 assay. (B) IC50 values of ISP I for the indicated BL cell lines.

**Supplementary Figure 2.** (A, B) Flow cytometric analysis of Carrimycin-induced (10 μM) apoptosis in Raji (A) and NAMALWA (Β) cells following co-treatment with 100 μM of the pan-caspase inhibitor Z-VAD-FMK for 24 hours. Representative flow cytometry plots are shown on the left, and the corresponding quantitative analysis is shown on the right. ****P* < 0.001, **** *P* < 0.0001. (C, D) Representative flow cytometry plots and quantitative analysis of apoptosis (Annexin V/PI staining) in Raji (C) and NAMALWA (D) cells treated with gradient concentrations of Carrimycin (5, 10, and 20 μM) for 48 hours. Representative flow cytometry plots are shown on the left, and the corresponding quantitative analysis is shown on the right.

**Supplementary Figure 3.** (A) Representative fluorescence microscopy images of ROS levels in Carrimycin-treated NAMALWA cells. (B) Flow cytometric analysis of Carrimycin-induced (10 μM) ROS levels in NAMALWA cells by the addition of 10 mM NAC. The left are representative flow cytometry plots, and the right is quantitative analysis. (C) Representative confocal immunofluorescence images (left) and quantitative analysis of the number of nuclear γ-H2AX foci per cell (right) in NAMALWA cells treated with vehicle or Carrimycin (20 µM) for 24 hours. **P* < 0.05, ** *P* < 0.01.

**Supplementary Figure 4.** (A, B) Cell viability analysis of Raji (A) and NAMALWA (B) cells co-treated with the optimal combined concentrations of Carrimycin (2.5 μM) and R406 (1 μM) for 24, 48, and 72 hours. (C, D) Apoptosis analysis in Raji (C) and NAMALWA (D) cells following 24 hours of co-treatment with Carrimycin (2.5 μM) and R406 (1 μM). For each cell line, representative flow cytometry plots (Annexin V/PI staining) are shown on the left, and the corresponding quantitative analysis is shown on the right. **** *P* < 0.0001.

**Supplementary Figure 5.** (A) 2D interaction map illustrating the specific network of hydrogen bonds, hydrophobic interactions, and van der Waals forces stabilizing ISP I within the TCF3 binding pocket. (B) Radius of gyration (Rg) of the complex over the 100 ns simulation trajectory, indicating sustained structural compactness. (C) Solvent-accessible surface area (SASA) of the complex over 100 ns, demonstrating stable solvent exposure with minor adaptations to accommodate the ligand. (D) Root mean square fluctuation (RMSF) of individual TCF3 amino acid residues, illustrating low local structural flexibility and high stability of the binding pocket. (E) Cellular Thermal Shift Assay (CETSA) in NAMALWA cells treated with DMSO or Carrimycin across a 52-76°C temperature gradient. Line graph quantification (right) demonstrates significant thermal stabilization of TCF3 by Carrimycin. (F) Drug Affinity Responsive Target Stability (DARTS) Assay in Raji (Left) and NAMALWA (Right) cells treated with different concentrations of Carrimycin (5-20 μM). (G) Cycloheximide (CHX, 100 µg/mL) chase assay in NAMALWA cells co-treated with DMSO or Carrimycin for 0 to 8 hours. (H) Western blot analysis of TCF3 protein expression in Raji (left) and NAMALWA (right) cells. Cells were treated with the proteasome inhibitor MG132 (5 µM) and Carrimycin (20 µM) for 6 hours. (Ι) Western blot analysis of TCF3 expression in BL cells treated with Carrimycin (20 µM) with or without the lysosome inhibitor Bafilomycin A1 (100 nΜ) for 6 hours. **P* < 0.05, ****P* < 0.001.

**Supplementary Figure 6.** (A) Body weight of NSG mice during intracranial xenograft treatment. The figure below shows the number of mice in the Control and Carrimycin group. (B) H&E staining of major organs (kidney, liver, and lung) from control and Carrimycin-treated intracranial xenograft mice. (C) IHC analysis of Ki-67, p-SYK, and TCF3 expression in intracranial xenografts. (D) Quantitative H-score analysis of IHC staining in subcutaneous and intracranial xenograft models (n=3 per group). **P* < 0.05, ** *P* < 0.01, ****P* < 0.001, **** *P* < 0.0001.
